# Host Coenzyme Q Redox State Is an Early Biomarker of Thermal Stress in the Coral *Acropora millepora*

**DOI:** 10.1371/journal.pone.0139290

**Published:** 2015-10-01

**Authors:** Adrian Lutz, Jean-Baptiste Raina, Cherie A. Motti, David J. Miller, Madeleine J. H. van Oppen

**Affiliations:** 1 AIMS@JCU, James Cook University, Townsville, Queensland, Australia; 2 Australian Institute of Marine Science, Townsville, Queensland, Australia; 3 Comparative Genomics Centre and Department of Molecular and Cell Biology, James Cook University, Townsville, Queensland, Australia; 4 ARC Centre of Excellence for Coral Reef Studies, James Cook University, Townsville, Queensland, Australia; 5 College of Marine and Environmental Sciences, James Cook University, Townsville, Queensland, Australia; 6 School of BioSciences, The University of Melbourne, Parkville, Melbourne, Victoria, Australia; Pennsylvania State University, UNITED STATES

## Abstract

Bleaching episodes caused by increasing seawater temperatures may induce mass coral mortality and are regarded as one of the biggest threats to coral reef ecosystems worldwide. The current consensus is that this phenomenon results from enhanced production of harmful reactive oxygen species (ROS) that disrupt the symbiosis between corals and their endosymbiotic dinoflagellates, *Symbiodinium*. Here, the responses of two important antioxidant defence components, the host coenzyme Q (CoQ) and symbiont plastoquinone (PQ) pools, are investigated for the first time in colonies of the scleractinian coral, *Acropora millepora*, during experimentally-induced bleaching under ecologically relevant conditions. Liquid chromatography-mass spectrometry (LC-MS) was used to quantify the states of these two pools, together with physiological parameters assessing the general state of the symbiosis (including photosystem II photochemical efficiency, chlorophyll concentration and *Symbiodinium* cell densities). The results show that the responses of the two antioxidant systems occur on different timescales: (*i*) the redox state of the *Symbiodinium* PQ pool remained stable until twelve days into the experiment, after which there was an abrupt oxidative shift; (*ii*) by contrast, an oxidative shift of approximately 10% had occurred in the host CoQ pool after 6 days of thermal stress, prior to significant changes in any other physiological parameter measured. Host CoQ pool oxidation is thus an early biomarker of thermal stress in corals, and this antioxidant pool is likely to play a key role in quenching thermally-induced ROS in the coral-algal symbiosis. This study adds to a growing body of work that indicates host cellular responses may precede the bleaching process and symbiont dysfunction.

## Introduction

Elevated seawater temperatures in conjunction with high solar irradiance disrupt the relationship between reef-building corals (Cnidaria: Scleractinia) and their dinoflagellate symbionts (*Symbiodinium* sp.) [1] and have been implicated in causing mass coral bleaching events [2–4]. Although the molecular events underlying the loss of *Symbiodinium* cells via exocytosis [5] and apoptosis [6] remain unclear, it is broadly accepted that coral bleaching is preceded by oxidative stress: the excessive formation of reactive oxygen species (ROS) which eventually overwhelm the antioxidant defence capacity of the symbiosis [7–9]. Initial impairment of photosynthesis is thought to increase ROS formation in the symbionts, leading to oxidative damage in the host, which then initiates bleaching [7]. Several potential primary damage sites have been identified in *Symbiodinium* during thermal stress, including photosystem II (PSII) reaction centres [10–12], antenna pigments [13], the Calvin cycle [14], and the thylakoid membranes [15]. Other evidence suggests that the primary site of thermal damage in *Symbiodinium* varies among coral species and symbiont types [16] which may explain some of the apparent contradictory results to date. In addition, there is increasing evidence suggesting that the cnidarian host plays a more significant role in the bleaching cascade than previously thought because thermal stress can compromise host cells prior to damaging the symbiont [17–20] and because bleaching can occur in darkness, independent of photosynthetically produced ROS [21]. Nonetheless, it is clear that the coral host has substantial antioxidant potential, indicating ROS scavenging during exposure to thermal and irradiance stress is essential in both symbiotic partners in order to prevent bleaching [22–26]. Hence, oxidative stress is likely to reflect an imbalance between the antioxidant capacity of both partners and the performance of the electron transport chains (ETC) of coral mitochondria and *Symbiodinium* chloroplasts [7].

As components of both antioxidant defence systems and the electron transport chains that generate ROS, the prenylquinones coenzyme Q (CoQ; ubiquinone) and plastoquinone (PQ) and their respective reduced (antioxidant) forms ubiquinol (CoQH_2_) and plastoquinol (PQH_2_) may play key roles in the bleaching response. These redox carriers play an integral role in electron transport (CoQ/CoQH_2_ in the mitochondrial ETC and PQ/PQH_2_ in the photosynthetic ETC) but also have important antioxidant functions within mitochondrial [27], cellular [28] and thylakoid [29] membranes. The reduced forms of these prenylquinones are highly effective lipid peroxidation chain breakers, and are involved in the regeneration of other antioxidants such as ascorbate and α-tocopherol [28, 30–32]. In addition, PQH_2_ is an effective singlet oxygen (^1^O_2_) quencher in chloroplasts [33, 34]. Consequently, shifts in the proportion of reduced to oxidised prenylquinones (%CoQH_2_; %PQH_2_) have been used to infer oxidative stress and ROS scavenging activity in plant models [30, 33, 35, 36].

Little is known about how the coral CoQ and symbiont PQ pools respond to hyperthermal stress. In a proof of concept study, Lutz et al. [37] demonstrated that the *Acropora millepora* CoQ and the *Symbiodinium* PQ pool redox states are maintained predominantly in their reduced forms (a prerequisite for antioxidant action), and acute heat-stress causes increased oxidation of the coral CoQ pool consistent with evidence that oxidative stress occurs in both host and symbiont [23, 24, 38]. However, due to the acute nature of the stress applied, it is unclear whether the observed oxidative shift was a consequence of metabolic failure, or whether the CoQ pool is sensitive to prolonged elevated temperature stress under more ecologically relevant conditions.

Here, quantitative liquid chromatography-mass spectrometry (LC-MS) was used to estimate the redox states of host CoQ and *Symbiodinium* PQ pools in colonies of the scleractinian coral *A*. *millepora* during experimentally-induced bleaching under ecologically relevant temperature conditions. The data on CoQ and PQ pool redox status, in combination with PSII photochemical efficiency, chlorophyll concentration and *Symbiodinium* density estimates were used to follow the effects of thermal stress on the state of the symbiosis over time.

## Materials and Methods

### Ethics Statement

All necessary permits were obtained for the described field studies. Specimens for this study were collected under permit number G09/30237.1, issued by the Australian Government’s Great Barrier Reef Marine Park Authority. The locations of sample collection are not privately-owned, and no endangered or protected species were collected.

### Reagents

All reagents, and the standards ubiquinone-9 (CoQ_9_) and ubiquinone-10 (CoQ_10_), were purchased from Sigma Aldrich (USA). Plastoquinone-9 (PQ_9_) was a kind gift from Professor Ewa Swiezewska from the Polish Academy of Sciences, Poland. All solvents used were HPLC grade (Mallinckrodt, Australia).

### Experimental design

Twelve *A*. *millepora* colonies roughly 50 cm in diameter containing type C2 *Symbiodinium* (ITS1 terminology, see below) were collected from Pelorus Island, Great Barrier Reef, Australia (18°33’ S/146°29’ E) in May 2010. Colonies were transferred to the Australian Institute of Marine Science (Townsville) and divided into a total of 24 fragments, each comprising approximately 25 branches. Fragments were arranged in eight indoor tanks in a balanced randomised block design, resulting in the allocation of twelve coral fragments (three per tank) to each of the control and thermal stress temperature treatments (27°C and 32°C, respectively). All tanks were continuously supplied with fresh, 1 μm filtered seawater at a rate of 1.5 L min^−1^ from 500 L reservoirs in a temperature-controlled room maintained at 27 ± 0.5°C (two reservoirs per treatment). Each reservoir was heated with two titanium heaters (3 kW) controlled by a CR1000 datalogger (Campbell Scientific) and a temperature sensor in the treatment tanks. All tanks were fitted with a small power head pump to maintain water movement and an air stone and pump to provide aeration. UV-filtered 400 W metal halide lights (BLV, Germany) were mounted above each tank and provided an average underwater light intensity of 350μmol photons m^−2^ s^−1^ (12:12 h light:dark cycle). The UV-filters were used to minimise UV-radiation-induced bleaching [39].

**Fig 1:**
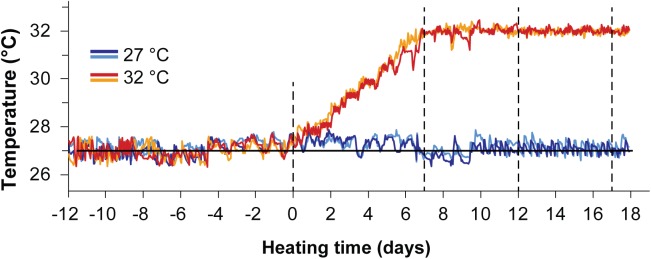
Temperature logger data for the experimental period. Thermal log of the four temperature sensors placed in heated (32°C) and control (27°C) seawater aquarium tanks for the duration of the experimental period. Two temperature sensors were used per treatment. Dashed lines indicate sampling time points.

The colony fragments were acclimated for two weeks prior to starting the experiment, then seawater temperatures in four tanks were ramped at a constant rate (0.7°C d^-1^) to 32 ± 0.5°C over seven days; the remaining four control tanks were maintained at 27°C for the entire duration of the experiment (Fig 1). The heat stress temperature was chosen to represent an ecologically relevant 1°C above the estimated local bleaching threshold of approximately 31°C for nearby Orpheus Island, Great Barrier Reef (18°35’ S/146°29’ E; ~31°C: [40]). Coral branches were sampled at four time points during the experiment: at the end of the acclimation period (*t* = 0 d), upon reaching the 32°C target temperature in the hyperthermal stress treatment (*t* = 7 d), and after five (*t* = 12 d) and ten days (*t* = 17 d) at 32°C. At each time point, coral nubbins (approximately 50 mm in length) were collected from each coral fragment after six hours of light (*n* = 12 in control and heat treatment, respectively) and from a subset of fragments after six hours of darkness (*n* = 9 in control and heat treatment, respectively). Samples were immediately snap-frozen in liquid nitrogen at time of collection to quench the PQ and CoQ pool redox states. Total sample size was optimised to ensure all samples could be processed in less than four weeks after sampling to ascertain the redox stability of the extracts.

### Photosystem II photochemical efficiency

Photosystem II (PSII) photochemical efficiency, expressed as maximum quantum yield ((F_M_−F_O_)/F_M_ = F_V_/F_M_) was measured daily with a Diving-PAM (Walz Gmbh, Germany) from three randomly chosen branches per coral fragment two hours before the start of the light cycle [41]. A 6 mm fibre optic probe was placed perpendicular to the surface at least 20 mm from the tip of the branch and 3 mm from the coral tissue surface (controlled via a rubber spacer) to obtain the measurements. Minimum fluorescence (F_O_) was measured using a weak pulsed measuring light (< 0.15 μmol photons m^−2^ s^−1^; gain = 3) and maximum fluorescence (F_M_) was measured upon application of a saturating pulse of light (> 4000 μmol photons m^−2^ s^−1^).

### Prenylquinone quantification

Coral nubbins for prenylquinone extraction were stored in liquid nitrogen for a maximum of 48 hours. Nubbins were extracted using a 1:1 mixture of isopropanol and ethyl acetate containing 0.1 μM CoQ_9_ (internal standard). Coral CoQ_10_ and *Symbiodinium* PQ_9_ pools were quantified by LC-MS using a slightly modified method of Lutz et al. [25]. In brief: prenylquinones were resolved using a Phenomenex Kinetex C18 column (150 mm × 4.6 mm, 2.6 μm particle size) on an Agilent 1100 series HPLC (Agilent, USA) coupled to a Bruker Esquire 3000 (Bruker Daltonics, USA). Absolute quantities of the prenylquinones were calculated from calibration plots obtained from standard compounds containing 0.1 μM CoQ_9_ (internal standard). CoQ and PQ redox states (%PQH_2_ and %CoQH_2_) were expressed as the proportion of reduced to total (oxidised + reduced) prenylquinone. Coral CoQ data could potentially be biased by CoQ of *Symbiodinium*; however, symbiont CoQ was not detected with the method applied here, either because *Symbiodinium* type C2 contains a different isoform than the host CoQ_10_ or because concentrations are below the detection limit [37].

### *Symbiodinium* densities

For *Symbiodinium* densities, one coral nubbin per coral fragment and time point was immediately processed at midday (*n* = 12 in control and heat treatment, respectively). Coral nubbins were airbrushed in individual plastic bags in 4 mL of 0.2 μm filtered seawater. The slurry was homogenised using a Turrax disperser (IKA, Germany) to break down aggregates and was centrifuged at 3000 g. The supernatant was removed and the pellet resuspended in 1 mL of 10% formalin in phosphate buffer saline (PBS). *Symbiodinium* cells were counted under a light microscope (eight technical replicates per sample) using a haemocytometer (depth 0.1 mm).

### Calculation of surface area and chlorophyll concentrations

Tissue remaining on the coral nubbins was removed by soaking in diluted commercial bleach (0.5% NaClO) overnight. Surface areas of the coral nubbins were determined using a wax dipping technique [42]. Chlorophyll concentrations (*a* and *c*_*2*_) were measured from aliquots of the prenylquinone extracts on a microplate reader (Powerwave, Bio-Tek Instruments, USA) [43] and determined using the equations presented in Ritchie et al. [44].

### *Symbiodinium* genotyping

The *Symbiodinium* genotypes were identified based on sequence differences in the nuclear ribosomal DNA internal transcribed spacer 1 (ITS1) region using single-strand conformation polymorphism (SSCP) analysis as described by van Oppen et al. [45]. Total coral and *Symbiodinium* DNA was extracted using a modified protocol [46] and the *Symbiodinium* ITS1 region amplified with fluorescently labelled Sym ITS1 PCR primers for SSCP analysis on non-denaturing polyacrylamide gels. The symbiont genotype was determined by comparison of manually scored gel images of known reference standards run in parallel with the samples [47]. SSCP profiles from all colonies were single bands identical to type C2 *Symbiodinium* (GenBank Accession AF380552) *sensu* van Oppen et al. [45].

### Transmission electron microscopy (TEM)

At each time point, one coral branch was sampled in four coral fragments per treatment, transferred directly into fixative (1.25% glutaraldehyde + 0.5% paraformaldehyde in 0.2 μm filtered seawater) and stored at 4°C until required. Fixed coral nubbins were decalcified in a formic acid:fixative mixture (1:3), with the solution changed every 12 h until complete dissolution of the skeleton. Three individual polyps per sample were postfixed in osmium and subsequently dehydrated with increasing concentrations of ethanol followed by dry acetone. Dehydrated samples were infiltrated in increasing concentrations of Araldite resin before being cured for 24 h at 60°C. Longitudinal sections, 90-nm thick, were collected on copper grids and imaged at 120 kV in a JEOL 2100 TEM.

### BLAST analysis

The *A*. *millepora* transcriptome [48], the *Acropora digitifera* genome [49] and the cnidarian protein and nucleotide database at NCBI were searched for enzymes involved in CoQ redox reactions. Homologue proteins and gene sequences were identified using BLAST (blastp, blastx, tblastx, tblastn) at http://blast.ncbi.nlm.nih.gov and http://marinegenomics.oist.jp, and the *A*. *digitifera* annotation available at http://bioserv7.bioinfo.pbf.hr/Zoophyte/index.jsp [50]. All identified sequences were assessed against the SwissProt database (http://www.uniprot.org/).

### Statistical analysis

Linear mixed models [51, 52] were applied to assess treatment effects using time (sampling day), treatment (control vs. heated) and the interaction as fixed effects and a random intercept for each coral fragment to account for repeated measures of the same colonies. F_V_/F_M_, %PQH_2_ and %CoQH_2_ data were power transformed; PQ concentration was log transformed. Model comparison was conducted using Akaike’s information criterion (AIC). Tank effects were non-significant (fixed) and redundant (random), and thus discarded to avoid overfitting in all models. First order autocorrelation covariate structure was determined as best model fit in all models. Multiple pairwise comparisons were corrected using the false discovery rate following Hochberg and Benjamini [53]. All statistical analyses were conducted using SPSS version 17.0.

## Results

**Fig 2:**
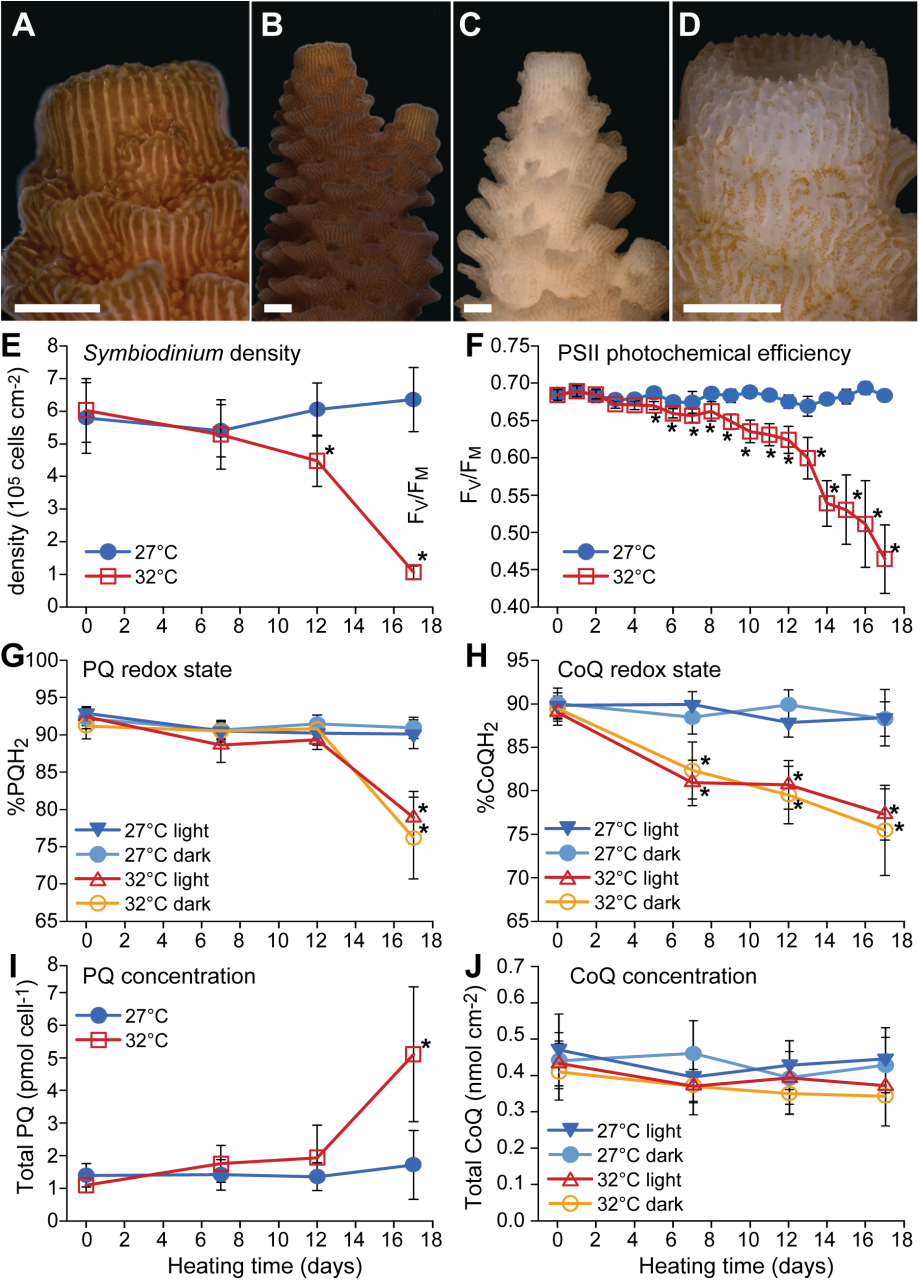
Effects of thermal stress on physiological parameters of the scleractinian coral *Acropora millepora*. Images of representative coral nubbins demonstrating the visual difference in *Symbiodinium* cell densities within *A*. *millepora* tissues under control (27°C) (A—B) and thermal stress (32°C) (C—D) conditions at day 17 (end of experiment). Scale bars = 1 mm. Thermal stress effects on (E) *Symbiodinium* density; (F) photosystem II photochemical efficiency; (G) plastoquinone (%PQH_2_) and (H) coenzyme Q (%CoQH_2_) pool redox states; (I) total plastoquinone concentration (PQ + PQH_2_) per *Symbiodinium* cell and (J) total coenzyme Q concentration (CoQ + CoQH_2_) per coral surface area over the course of the experiment. All data points are means ± 95% CI; * indicate significant differences between control and treatment at *p* < 0.05; *n* = 6–12 (see Table 1 for details).

**Fig 3:**
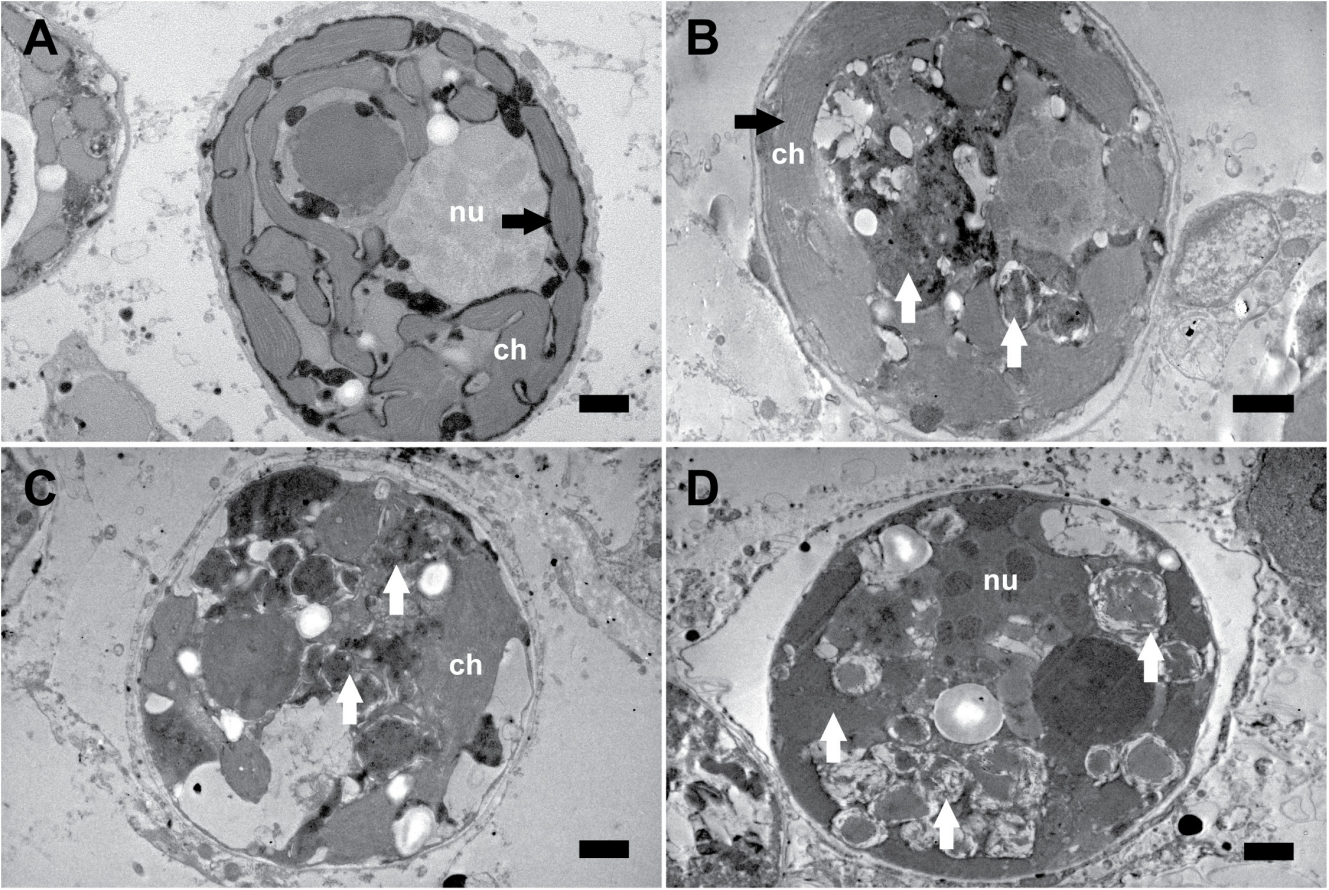
Representative transmission electron micrographs documenting the effects of thermal stress on the internal structure of endosymbiotic *Symbiodinium* cells within tissue of *Acropora millepora*. (A) *Symbiodinium* exposed to 27°C showing intact organelles and thylakoid membranes (black arrow). (B) First signs of degraded internal structures in some *Symbiodinium* cells after 7 days of heat stress (white arrows). Note the intact structure of the thylakoid membranes (black arrow). (C and D) *Symbiodinium* exposed to 32°C revealing degraded internal structures (white arrows). Scale bars, 1 μm; ch, chloroplast; nu, nucleus.

*A*. *millepora* colony fragments exposed to hyperthermal stress (32°C) showed clear symptoms of bleaching, when compared to ambient (27°C) treatment controls (Fig 2A–2D). In the thermal stress treatment group, *Symbiodinium* cell densities were reduced by 25.7% after five days and by 82.4% at the end of the experiment (Fig 2E). No significant changes were observed in cellular chlorophyll concentrations (*a* and *c*_2_) during the experiment (mean = 29.6 ± 4.5 pg cell^−1^; *p* = 0.46). Mortality was low; of the 24 colony fragments used, only two of the 12 exposed to thermal stress showed signs of necrosis, patchy tissue sloughing and algal overgrowth. No further data were collected for these fragments after day 13 and 15, respectively, when symptoms of mortality were first observed. PSII photochemical efficiency (F_V_/F_M_ ± 95% confidence interval (CI)) remained stable in control colonies (mean = 0.68 ± 0.1) but declined markedly in the 32°C treatment group concomitant with the loss of *Symbiodinium* cells after day 9 (Fig 2F; *p* < 0.001; Table 1). The declining trend of F_V_/F_M_ was observable from the third day after heating commenced; however, F_V_/F_M_ did not differ significantly from control samples until day five (t-test; F_1,278_ = 10.683; *p* = 0.0012). TEM images showed no impact on the *Symbiodinium* thylakoid membrane or cell wall structure in the first seven days of the experiment; however, disintegrated internal organelles were observed in 7% of the cells examined (total 1502). After five days exposure to 32°C (*t =* 12 d), all of the remaining *Symbiodinium* cells exhibited both structurally compromised thylakoid membranes and widespread disintegration of organelles in the cytoplasm (Fig 3). While damage to internal structures was apparent, cell walls appeared intact and no fragmented symbiont cells were observed.

**Table 1:** Linear mixed model testing for differences in temperature treatments (27°C = control; 32°C = stress) during a hyperthermal bleaching experiment of *Acropora millepora* containing *Symbiodinium* type C2.

		Factor								
		Treatment			Time^a^			Treatment × time		
	*n*	df	*F*	*p*	df	*F*	*p*	df	*F*	*p*
F_V_/F_M_	12^b^	22.0	104.2	**<0.001**	201.8	15.8	**<0.001**	201.8	13.8	**<0.001**
%PQH_2_ (light)	12^b^	22.1	12.0	**0.002**	48.8	21.6	**<0.001**	48.8	6.3	**0.001**
%PQH_2_ (dark)	9^c^	19.6	10.0	**0.005**	41.1	9.6	**<0.001**	41.1	5.7	**0.002**
Total PQ cell^-1^	6	9.0	5.5	**0.043**	17.9	7.7	**0.002**	17.9	5.5	**0.008**
Total chlorophyll cell^-1^	6	10.4	0.6	0.46	20.8	0.2	0.88		n/a	
%CoQH_2_ (light)	12^b^	22.1	24.5	**<0.001**	41.2	17.0	**<0.001**	41.2	14.8	**<0.001**
%CoQH_2_ (dark)	9^c^	15.9	22.5	**<0.001**	33.6	12.8	**<0.001**	33.6	6.3	**0.002**
Total CoQ cm^-2^ (light)	12^b^	22.3	0.9	0.36	42.0	3.5	**0.023**	42.0	0.2	0.91
Total CoQ cm^-2^ (dark)	9^c^	15.9	1.7	0.21	34.4	2.0	0.135		n/a	

CoQ, coenzyme Q; %CoQH_2_, coenzyme Q pool redox state; F_V_/F_M_, maximum quantum yield; PQ, plastoquinone; %PQH_2_, plastoquinone pool redox state.

^a^ F_V_/F_M_ was measured daily (18 time points), other measurements at four time points.

^b,c^ replication number given is for the full set. Due to dropouts, for the last time point *n* = 10 (^b^) and *n* = 8 (^c^).

*p*-values significant at α < 0.05 are highlighted in boldface.

The *Symbiodinium* PQ pool was predominantly reduced at the start of the experiment, and remained approximately constant during the first twelve days of heat stress (from *t =* 0 h to 12 d: mean = 90.8 ± 1.4% in light and 87.3 ± 4.2 in dark); note that the PQ redox state (%PQH_2_ ± 95% CI) did not differ significantly between the light and dark periods (Fig 2G; Table 1). However, the heat stressed colonies exhibited a highly significant 12% (light) and 11% (dark) decline in PQH_2_ at the end of the experiment (at *t* = 17 d, mean = 78.9 ± 3.5% and 76.2 ± 5.1%; respectively; *p* < 0.002). When normalised per *Symbiodinium* cell, this decline in PQH_2_ coincided with an apparent five-fold increase in total PQ concentration (PQ + PQH_2_) from 1.49 ± 0.23 pmol cell^−1^ (mean, *t =* 0 d to 12 d) to 5.11 ± 2.06 pmol cell^−1^ at *t* = 17 d (Fig 2I; *p* = 0.008) in the heat treatment.

Whereas the redox state of the PQ pool remained essentially stable for 12 days, the coral CoQ pool (%CoQH_2_ ± 95% CI) became oxidised more rapidly in response to the hyperthermal stress. CoQH_2_ declined from 89.4 ± 1.0% to 80.9 ± 2.6 in the light, and from 89.7 ± 1.1% to 82.3 ± 3.3% in the dark, within the first seven days of the experiment (i.e. at the end of the heating phase from 27°C to 32°C; Fig 2H; *p* < 0.002). In the 32°C treatment group, %CoQH_2_ continued to decline steadily over the next ten days to 77.3 ± 2.9% (light) and 75.4 ± 7.9% (dark). As in the case of the plastoquine pool, light and dark estimates of %CoQH_2_ did not differ significantly. Likewise, total CoQ (CoQ + CoQH_2_; normalised per coral surface area) did not differ between control and heat stress, remaining stable throughout the experiment *(* *Fig* 2 *J; Light*: *mean = 0*.*41 ± 0*.*03 nmol cm* ^*−2*^ *; p = 0*.*36; Dark*: *mean = 0*.*40 ± 0*.*03 nmol cm* ^*−2*^ *; p = 0*.*21)*.

## Discussion

### Coenzyme Q pool redox state

The results presented here demonstrate that the *A*. *millepora* host CoQ redox state is sensitive to hyperthermal stress, exhibiting an overall 13% decline of CoQH_2_ in response to the stressor (Fig 2H). This oxidative shift was not caused by an increase of *de novo* synthesised CoQ, as the total CoQ concentration (CoQ + CoQH_2_) did not increase during the heat stress. Oxidation of the CoQ pool occurred early in the thermal stress treatment, upon reaching 32°C (after seven days with a daily temperature increase of approximately 0.7°C per day) and prior to any measurable loss of *Symbiodinium* cells from the host tissue. Oxidation of the CoQ pool occurred before a major decline in PSII photochemical efficiency was observed, i.e., while the effects of the hyperthermal stress on the *Symbiodinium* photosynthesis apparatus were still limited (F_V_/F_M_ > 0.65). Moderate irradiance levels (350 μmol photons m^−2^ s^−1^) were used in order to avoid major light stress concomitantly with the applied hyperthermal stress [2, 54, 55]. Maximum daily irradiance at 1–3 m depths regularly exceeds 1000 μmol photons m^−2^ s^−1^ for nearby (< 25 km), equally turbid Great Palm Island waters [56]. The results therefore indicate that the *A*. *millepora* CoQ pool is oxidised significantly in response to hyperthermal stress in the absence of strong light exacerbating ROS leakage from the symbiont [57, 58]. Although a contribution of photosynthetically derived ROS to the observed CoQ pool oxidation cannot be discounted, the results presented here add to a growing body of work that indicates host cellular responses may precede the bleaching process and symbiont dysfunction [5, 17–20, 59]. In addition, other thermal stress-related responses such as transcriptional and physiological changes that were not measured here are expected to occur in both coral symbiosis partners prior to host CoQ pool oxidation. For example, other reported early changes include a reduction in epithelial tissue, signs of increased apoptosis in the gastrodermis, and changes to the transcriptome, which have been associated with an upregulation of chaperone and antioxidant defence genes alongside transcriptional changes that, by analogy to vertebrate models, are assumed to be linked to apoptosis [18, 25, 26, 60–63]. It should also be noted that the heat/light sensitivity of the photosynthetic apparatus varies among different symbiont types and that this affects host sensitivity to bleaching [64–66]; however, bleaching susceptibility differs widely among different coral genera despite often hosting the same *Symbiodinium* types [67–69]. Considering this, the results presented here require confirmation in other symbiont-host associations prior to postulation of a generalized physiological response during the bleaching cascade. Nonetheless, the oxidative shift in the CoQ redox state observed here is among the earliest known metabolic changes in the coral partner in response to a realistic temperature level.

#### Coenzyme Q pool redox state regulation

**Fig 4:**
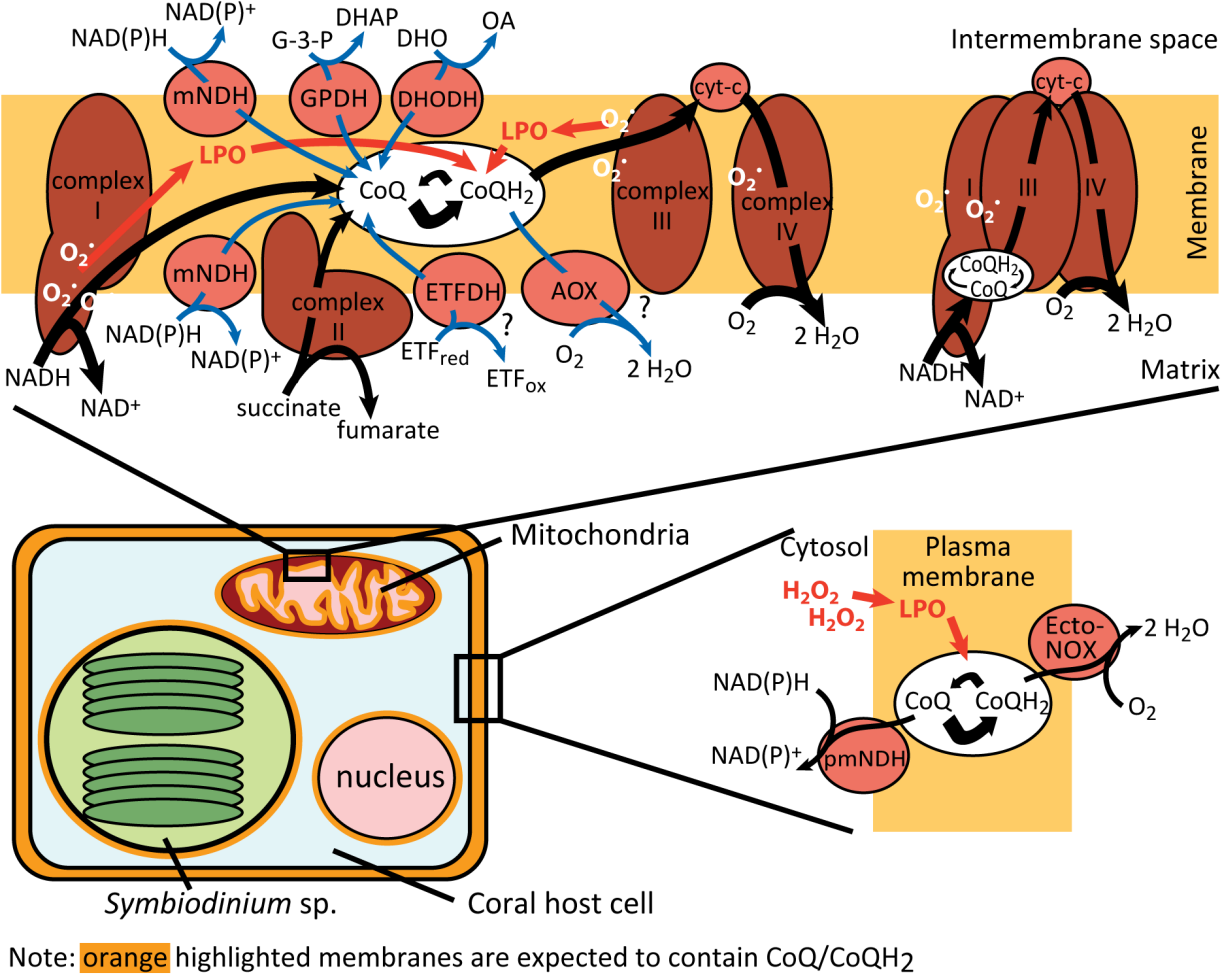
Schematic diagram of electron transfer reactions using the coenzyme Q (CoQ) pool in the coral mitochondrial and plasma membrane electron transport. Respiratory “linear” electron flows (black arrows) proceed from NADH in the mitochondrial matrix to H_2_O via the CoQ pool and the enzyme complexes I, II, III, and IV, forming ubiquinol (CoQH_2_) as an intermediary product. The electron flows via complexes I, III and IV occur (mostly) via tunnelling or micro-diffusion of CoQ/CoQH_2_ in I-II-IV supercomplexes rather than via the larger mobile CoQ pool [72]. “Non-linear” electron flows (dark blue arrows) proceed from electron donors (e.g. NAD(P)H) via several quinone dehydrogenases to the CoQ pool, and to H_2_O from CoQH_2_ via AOX. Plasma membrane electron transport occurs from NAD(P)H to H_2_O via one or more type of NAD(P)H-CoQ reductases, the plasma membrane CoQ pool and Ecto-NOX. CoQH_2_ ROS scavenging occurs continuously in O_2_ metabolism primarily via chain breaking of lipid peroxidation (LPO) caused by O_2_^•−^ and H_2_O_2_. Abbreviations: AOX, alternative oxidase; cyt-c, cytochrome c; DHAP, dihydroxyacetone phosphate; DHO, dihydroorotate; DHODH, dihydroorotate dehydrogenase; Ecto-NOX, external quinone oxidase; ETF_red/ox_, reduced/oxidised electron-transferring-flavoprotein; ETFDH, electron-transferring-flavoprotein dehydrogenase reduced/oxidised; Ecto-NOX, external quinone oxidase; GPDH, glycerol-3-phosphate dehydrogenase; G-3-P, glycerol-3-phosphate; H_2_O_2_, hydrogen peroxide_;_ LPO, lipid peroxidation; pmNDH/mNDH, plasma membrane/mitochondrial NAD(P)H dehydrogenases; OA, orotate; O_2_^•−^, superoxide.

CoQ/CoQH_2_ is present (in varying quantities) in all intracellular membranes of every animal with the highest concentrations found in the mitochondrial membranes at the primary site of ROS production [32, 70]. In eukaryotes, CoQ redox processes are relatively complex (Fig 4; for relevant enzymes identified in *Acropora* sp. see S1 Table). Within mitochondrial membranes, CoQH_2_ is continuously regenerated by the respiratory chain (complex I, II and alternative NAD(P)H dehydrogenases) [71] and other mitochondrial enzymes (glycerol-3-phosphate dehydrogenase, electron-transferring flavoprotein dehydrogenase, dihydroorotate dehydrogenase; [72]). In other membranes, several enzymes catalyse CoQ reduction including a NADH-cytochrome b_5_ reductase [73] and a distinct, unresolved NADPH-CoQ reductase [74]. Interestingly, a cytosolic NAD(P)H:quinone reductase (NQO1; formerly DT-diaphorase) [75]–the most studied CoQ reducing enzyme–appears to be absent in cnidarians along with other NQO genes [76].

In contrast to the reduction of the CoQ pool, CoQH_2_ is oxidised by direct interaction with ROS, in particular lipid peroxyl radicals and the lipid peroxidation initiating perferryl radicals (Fe_3_O_2_^•−^) found in all membranes [28], by complex III and alternative oxidases (AOX) of the mitochondrial ETC [77], and by the external oxidases of the plasma membrane electron transport (Ecto-NOX; [78]). Considering the known CoQ pool redox mechanisms of other animals, the oxidative shift in the CoQ redox state in *A*. *millepora* can therefore be attributed to: (1) an increase in ROS scavenging by CoQH_2_; (2) a decline in net CoQ reduction by the mitochondrial ETC; (3) extra-mitochondrial pathways; or 4) any combination of these processes.

#### Coenzyme Q pool reactive oxygen species scavenging in corals

Attributing the thermal stress-induced oxidative shift in CoQ redox state of *A*. *millepora* to a specific physiological mechanism is difficult, primarily because current understanding of CoQ functions in the coral-*Symbiodinium* symbiosis is very limited and existing methods cannot distinguish between functionally and spatially different CoQ pools present in different organelles [79]. Theoretically, a net decline in CoQ reduction caused by the mitochondrial ETC or extra-mitochondrial pathways are conceivable by postulating a decline in CoQ reducing or an increase in CoQH_2_ oxidising enzyme activities; however, no such direct impact of thermal stress on the CoQ pool has been demonstrated so far. In particular, the emerging consensus that the complexes I-III-IV occur mostly as supercomplexes further complicates attributing shifts in the CoQ redox state to a specific location in the mitochondrial ETC as electron transfer in these supercomplexes appears to occur via tunnelling or microdiffusion of CoQ/CoQH_2_ rather than via a mobile CoQ pool in mitochondrial membranes [72].

Short term heat stress in the bleaching model *Aiptasia* has been reported to cause the degradation of host mitochondria prior to symbiont impairment and to lead to the downregulation of genes associated with ATP production and electron transport at the site of, and downstream from, cytochrome c [19]. However, the report did not include any genes upstream of complex III (Fig 4), thus there is no indication that the CoQ pool reducing side of the mitochondrial ETC was affected. A recent transcriptional analysis provided further evidence of the thermal stability of complex III gene expression in *A*. *millepora* [80] but analyses at the enzyme activity level in cnidarians remain outstanding. The visible, pre-bleaching mitochondrial damage in *Aiptasia* [19] would be expected to incapacitate the mitochondrial ETC and potentially lead to CoQ oxidation due to a decline in electron flux to the pool; however, there is currently no data available to lend support to such a model. On the other hand, an inefficient mitochondrial ETC is likely leading to the increased formation of ROS [8, 59, 81], in particular during daytime hyperoxia [24, 82]. Thus, even though it is possible that any components of the CoQ pool redox mechanisms are affected by thermal stress, attributing the oxidative shift in the CoQ redox state to increased CoQH_2_ ROS scavenging in response to hyperthermal stress currently provides the most parsimonious explanation.

In mammals, oxidative shifts in the CoQ pool redox state have been observed in a variety of pathological conditions that are associated with oxidative stress [35, 36, 83]. These oxidative shifts are understood to result from an increasingly challenged antioxidant defence [28]. In cnidarian-*Symbiodinium* symbioses, it has been repeatedly demonstrated that the cnidarian host reacts to thermal stress and high light by increasing its antioxidant activities, which indicates an increased requirement to detoxify ROS in the host tissues [7, 38, 54, 84–86]. ROS formation also occurs in aposymbiotic cnidarians upon exposure to light and elevated temperatures, although in symbiosis, the hyperoxia caused by algal photosynthesis aggravates the coral’s innate ROS formation because it increases relative to oxygen concentration [24, 59, 87–91]. Bleaching in symbiotic cnidarians can also be triggered in the absence of photosynthetically produced ROS by thermal stress in darkness [21]. It is not yet understood what role non-photosynthetically produced ROS play in this dark-bleaching; however, mitochondria would appear to be the most likely origin for these ROS. Mitochondria (the primary source of ROS in animals) are the location of the highest cellular CoQ/CoQH_2_ concentration in eukaryotes [28, 78]. Here, superoxide (O_2_^•−^) and other ROS are generated by enzymes involved in the ETC, particularly the NADH dehydrogenase of complex I, and the interface between the CoQ pool and complex III [91, 92]. The co-localisation of the ROS producing respiratory ETC and the CoQ pool within mitochondrial membranes is likely an important factor in the high antioxidant effectiveness of CoQH_2_ [28, 93]. In addition to ROS generation by the coral, ROS leakage from *Symbiodinium* probably exceeds the host’s innate ROS generation [24, 57, 58]. Moreover, impaired or damaged photosynthetic ETC may further increase ROS formation and, ultimately, ROS leaking into the host [7, 8, 94]. The expulsion of *Symbiodinium* cells by the coral host has therefore been regarded as a protective mechanism: the coral prevents further ROS leakage from *Symbiodinium* into host cells by removing the primary source of ROS production and also by reducing tissue hyperoxia during daylight exposure [95, 96]. The CoQ pool likely provides an early line of antioxidant defence because ROS leaking from *Symbiodinium* cells would need to cross the host-derived symbiosomal membrane, which like all animal membranes is expected to contain CoQ/CoQH_2_ [32, 70]. Nonetheless, it would be expected that an increase in *Symbiodinium* cellular ROS concentrations to a point where leakage into the host occurs would manifest as a distinct decline in PSII photochemical efficiency. However, a major decline in F_V_/F_M_ was only observed here after significant CoQ pool oxidation had already occurred (Fig 2F and 2H). This suggests that ROS leakage is unlikely to be a major contributing factor to the initial oxidation of the CoQ pool, although this cannot be ruled out in the later stages where a major decline in F_V_/F_M_ was observed.

### Plastoquinone pool redox state

In contrast to the host CoQ pool redox state, the *Symbiodinium* PQ redox state remained stable until the point at which PSII photochemical efficiency was severely impaired and coral nubbins were distinctly bleached (Fig 2C and 2D). The observed initial stability of the PQ redox state, despite hyperthermal stress, is consistent with short-term acute heat stress results [37]. By analogy with high light stress [29, 97], the oxidative shift due to hyperthermal stress could be caused by increased ROS scavenging of PQH_2_ within *Symbiodinium* chloroplasts or changes in photosynthetic ETC such as increased plastid terminal oxidase activity. PQH_2_ is a highly effective quencher of ^1^O_2_ [33, 34] and, like CoQ, acts as a lipid peroxidation chain breaker either directly or via the regeneration of α-tocopherol [30, 31]. Even though irradiance was maintained at a moderate level during the experiment described here, the applied temperature stress caused chronic photoinhibition of PSII, which is commonly reported in coral bleaching experiments (e.g. [14, 15, 98]) and a known indicator of ROS formation within the photosynthetic ETC [99].

A five-fold increase in PQ pool concentrations was recorded concomitantly with the observed PQ pool oxidation (Fig 2I). Newly synthesized PQ is predominantly in the reduced form (PQH_2_, not PQ) [97], thus the observed oxidative shift in the PQ pool at this stage should be the result of increased non-enzymatic formation of PQ from PQH_2_ after its interaction with ROS, which are increasingly generated by a thermally damaged photosynthetic ETC [7, 14, 81, 94]. In plants and algae, a considerable proportion of the PQ pool is associated with the chloroplast plastoglobuli which are thought to act as PQH_2_ reservoirs [100, 101]. Consequently, the size of the PQ pool increases when plants and algae are exposed to conditions that induce the formation of ^1^O_2_, such as high light exposure [29, 33, 97]. Accordingly, the increase in the total PQ pool observed here could be seen as a cellular protective mechanism against the oxidative stress caused by the increasingly impaired photosynthetic ETC. The lack of compromised cell walls in the TEM images (Fig 3) implies that this increase is unlikely to be the result of a normalisation artefact: i.e. loss of structurally compromised cells during the extraction procedure, thus underestimating cell counts. Nonetheless, the concomitant loss of internal cellular structure and the highly compromised state of thylakoid membranes at this time point indicate a need for further experimental work before a definitive protective mechanism can be attributed to *de novo* synthesised (reduced) PQ during bleaching.

## Conclusions

This study demonstrated that hyperthermal stress in *A*. *millepora* was associated with oxidation of the coral host CoQ pool redox state. This oxidation occurred prior to any measurable loss of *Symbiodinium* cells from the host and major decline in PSII photochemical efficiency. Thus the oxidation of CoQ pool redox state is among the earliest known impacts of hyperthermal stress on the cellular chemistry of the coral host and adds to a growing body of work that indicates host cellular responses may precede the bleaching process and symbiont dysfunction. Furthermore, the *Symbiodinium* PQ pool redox state remained unaffected by hyperthermal stress until PSII photochemical efficiency was severely impaired. At this stage, the PQ pool exhibited a five-fold increase in concentration and a distinct oxidative shift.

## Supporting Information

S1 Table Enzymes involved in coenzyme Q pool redox reactions identified in the *Acropora digitifera* genome^a^, the *Acropora millepora* transcriptome^b^ and the EST sequences deposited at GenBank^c^. (PDF)
