# Supplementary material for: Host Coenzyme Q Redox State Is an Early Biomarker of Thermal Stress in the Coral Acropora millepora
Source: PLoS One. 2015 Oct 1;10(10):e0139290. doi: 10.1371/journal.pone.0139290 (PMC4591267; doi:10.1371/journal.pone.0139290)
Supplement: S1 Table — (PDF) [file pone.0139290.s001.pdf]

S1 Table. Enzymes involved in coenzyme Q pool redox reactions identified in the *Acropora digitifera* genome[a], the *Acropora millepora* transcriptome[b] and the EST sequences deposited at GenBank[c].

annotation	acronym	EC number	GO ID	*A. digitifera* protein ID	e-value	*A. millepora* genebank acc.
**CoQ reducing enzymes**						
glycerol-3-phosphate dehydrogenase	GPDH	EC 1.1.5.3	GO:0052590	aug_v2a.03411	3E-97	GO000270 JR989701
				aug_v2a.21935	3E-76	
				aug_v2a.03412	2E-39	
electron-transferring flavoprotein dehydrogenase	ETFDH	EC 1.5.5.1	GO:0004174	aug_v2a.07429	0E+00	GO004340 GO000415
				aug_v2a.17786	0E+00	JR978217 JR974781 JR984902 JR977609
dihydroorotate dehydrogenase	DHODH	EC 1.3.5.2	GO:0004152	aug_v2a.06198	4E-146	DY580717 JR981972 JT005565
NADH-cytochrome b_5 reductase	pNDH	EC 1.6.2.2	GO:0004128	aug_v2a.00893	9E-62	JT000401 JR980401 JR998872
				aug_v2a.12166	9E-61	
				aug_v2a.07042	7E-63	
alternative NAD(P)H dehydrogenase	NDH	EC 1.6.99.3	GO:0003954	aug_v2a.16501	1E-119	JR986898 JR984254
**CoQ oxidising enzymes**						
alternative oxidase 1, mitochondrial	AOX	EC 1.10.3.11	GO:0009916	aug_v2a.07092	2E-28	DY587694 JR988887 JT002555 JR978016
plasma membrane external oxidase	Ecto-NOX	-	-	aug_v2a.03517	5E-44	JT014973

Homologue proteins and gene sequences were identified using BLAST searches (http://ncbi.nlm.nih.gov; http://marinegenomics.oist.jp) and the *A. digitifera* annotation available at Zoophyte (http://bioserv7.bioinfo.pbf.hr/Zoophyte/index.jsp). E-values are for the top annotated BLAST hit to the SwissProt database. Not listed are the subunits of the main enzyme complexes in the respiratory chain (complexes I-II-II).

[a]Shinzato C, Shoguchi E, Kawashima T, Hamada M, Hisata K, et al. (2011) Using the Acropora digitifera genome to understand coral responses to environmental change. Nature 476: 320-323.

[b]Moya A, Huisman L, Ball EE, Hayward DC, Grasso LC, et al. (2012) Whole transcriptome analysis of the coral Acropora millepora reveals complex responses to CO2-driven acidification during the initiation of calcification. Mol Ecol 21: 2440-2454.

[c]Benson DA, Karsch-Mizrachi I, Lipman DJ, Ostell J and Wheeler DL (2005) GenBank. Nucleic Acids Res 33: D34-D38.
